# Design and evaluation of an image-dissector-based derivative spectrophotometer

**DOI:** 10.1155/S1463924686000044

**Published:** 1986

**Authors:** Timothy A. Nevius, Harry L. Pardue

**Affiliations:** Department of Chemistry Purdue University West Lafayette Indiana 47907 USA


**Journal of Automatic Chemistry**/*Journal of Clinical Laboratory Automation*, **Volume 8, Number 1 (January–March 1986), pages 12–17**

### Design and evaluation of an image-dissector-based derivative spectrophotometer

**Timothy A. Nevius and Harry L. Pardue**

*Department of Chemistry, Purdue University, West Lafayette, Indiana 47907, USA*

Several authors have reviewed the advantages of derivative spectroscopy in discriminating overlapping spectra in the quantitation of components in mixtures, and in resolving spectral information from background information [1 and 2]. Methods used to generate derivative spectra have included mechanical modulation of the wavelength dispersion optics [2]. This technique has the advantage of minimizing the effects of such time-dependent variables as source and detector drift. The disadvantages are that the amplitude of the wavelength modulation critically effects the sensitivity, linearity, and spectral resolution of the derivative measurement. An earlier report [3] from this laboratory described two derivative spectrophotometers which were based on vidicon imaging detectors. These instruments generated derivative signals by modulating the scanning electron beam of the vidicon and using a lock-in amplifier to monitor the first, or second, harmonic of the resulting signal to obtain first- or second-derivative spectra.

This paper describes an alternative approach in which derivative spectra are computed from intensity versus wavelength data recorded in a conventional scanning mode. A high-resolution image dissector camera [4] is used to monitor multiple wavelengths simultaneously and resulting signals are stored in computer memory. Digital methods are then used to compute derivative signals from the intensity data.

The theory of digital filtering and differentiation is described in the literature [5]. The resulting pseudoderivatives can be related to the concentration of a sample through Beer's Law, such that the quantities $(dI/d\lambda)/I$ and $(d^2I/d\lambda^2)/I$ (where $I$ is intensity and $\lambda$ is wavelength) are proportional to concentration. The basic approach in this instrument is to perform as much of the signal processing as possible by manipulating the raw intensity versus wavelength data in a minicomputer. The wavelength modulators and lock-in amplifiers used earlier [1–3] are replaced entirely by computer programs. Digital filtering is more flexible, and generally more effective, than the analogue techniques. The penalty of the digital techniques is that they are much slower than the analogue devices they replace. This is often an acceptable compromise, although it is desirable to optimize the programs for a fast execution time [6]. There is a tendency for the derivative process to enhance high-frequency noise [2] so there is a great deal of effort spent filtering and smoothing signals. Also, the wide flexibility of digital filters leaves ample opportunity for improper use that can lead to signal distortion and care must be exercised to avoid this.

### Experimental

*Instumentation*

*General:* The instrument system consists of a light source, suitable optics, a grating polychromator, and image-dissector camera, control/detection circuitry, and a computer. All optical components are mounted on an optical bench and the grating polychromator enclosure is securely bolted to the face-plate of the image dissector (ID) to ensure stability. Each part of the system is discussed in detail below.

*Detector:* The image dissector (Model 658A Optical Data Digitizer [ODD], EMR Photoelectric, Princeton, New Jersey, 08540, USA) is a high-resolution non-integrating detector, with a spectral response from 140 to 850 nm and a peak spectral response in the blue region. In this sensor, the optical image is transformed by a photoelectric cathode into an electrical signal, which is then magnetically focused onto a plate with an aperture at the centre. Magnetic deflection positions the desired $x$–$y$ location of the photocathode at the aperture. Electrons passing through the aperture are multiplied by a discrete-state electron multiplier. The resulting signal is an analogue of the original light illumination.

The image on the face of the detector is 28 mm square, and the detector is capable of resolving 2048 by 2048 pixels. The detector can randomly address 4096 $x$ locations at each of 4096 $y$ locations, with a nominal addressing time of 2 µs per address.

*Light sources:* For near ultra-violet studies, the light source used was a xenon arc lamp (Model EP102, Optical Radiation Corporation, Azusa, California 91702, USA) operated at 250 W. For studies in the visible region, the source was a tungsten-halogen lamp (No. 2331, General Electric Company, Cleveland, Ohio 44112, USA) powered by a regulated supply.

*Optics:* The polychromator (UFS-200, SA Instruments, Inc., Metuchen, New Jersey 08840, USA) is a flat-field type designed specifically for multiwavelength detectors. It produces a flat focal plane image located about 10 mm outside the housing. The grating used is a type III concave holographic grating with 175 grooves/mm. The theoretical resolution of the spectrograph obtainable with a 5-µm slit width is 1·2 nm. The 50-µm slit width used in this study yielded resolution of 5 nm. A 10-mm quartz cuvette was used for a sample cell. Quartz lenses focused light through the sample cell onto the entrance slit of the dispersion optics.

*Controller:* The controller serves as an intelligent interface between the processor and the image dissector. Its function is to automate some of the procedures involved in scanning the detector. The controller can provide either a raster scan of the entire face of the detector, or it can repetitively scan a single line or group of lines. The controller also operates the analogue-to-digital converter. A maximum scan rate of 180 lines/s is possible with the image dissector. The rate is limited by the slew rates of the scanning circuitry in the opitical data digitizer. The actual scan rate is limited by the speed of the PDP 12 to 24 lines/s. The computer is required to accept a data value, perform a double precision addition if ensemble averaging is used, and store the result in a buffer. The image dissector is operated only by the controller in this mode, which maximizes the scanning speed by relieving the processor of the task of controlling the camera.

It is also possible to control the image dissector directly from the computer, bypassing the controller, in order to allow random access to any pixel on the face of the detector. In this mode, the computer provides separate $x$ and $y$ co-ordinates and issues a convert command to the analogue-to-digital converter, in addition to averaging and storing the resulting data. This additional overhead limits the maximum scan rate to six lines/s.

*Computer:* The computer (PDP 12/30, Digital Equipment Corporation) is configured with a video terminal, a line-printer, a high-resolution plotter and a storage oscilloscope. Mass storage consists of a 2 megabyte disk and nine-track tape. The spectrophotometer can be considered as another peripheral connected to the computer communication bus.

*Software:* Programs written to control the image dissector are grouped into three broad categories: data acquisition, data processing, and data display. The programs are structured so that all intermediate data files have a common format in order to maximize flexibility in processing options. The output of any program can easily be used as the input to any other program.

The purpose of the data-acquisition routine is to acquire a data file of intensity wavelength. The standard double-precision data-acquisition program (DPDAQ) can ensemble average up to 4096 scans of the detector at a data density of 512 points/scan. The results are stored as 24 bit double-precision integers, or optionally converted to floating-point numbers with 24 bit mantissas. The maximum scanning rate is a least 24 scans/s in this mode. A random-access data-acquisition program (RANDAO) is provided to enable non-linear scanning modes. As noted earlier, the increased overhead of this program limits the maximum scanning rate to six scans/s.

The data-processing routines comprise all of the steps necessary to generate absorbance versus wavelength, or the derivative versus wavelength, information from the original intensity versus wavelength data. Programs are provided to perform noise filtering (SMOOTH), dark-current correction (STRCT), generation of first derivatives (GOLAY), generation of second derivatives (DTWO), or calculation of absorbance (ABSORB). In addition to these, these are several special data-processing routines that provide the same general capabilities, but which are optimized to achieve desired trade-offs between noise and distortion.

The data display routines convert the data files to graphical or tabular formats. A file can be displayed as an $x$–$y$ graph on a storage oscilloscope (TV) or plotted on paper (INTPLT). It is occasionally necessary to see the numerical data via a program that prints a data file (FILDMP).

There is a lot of flexibility inherent in this software system. In a typical experiment, a spectrum would be acquired with DPDAO, filtered with SMOOTH, corrected for dark current with STRCT, derivatized with GOLAY, and displayed on the storage oscilloscope with TV. At this point the operator can evaluate the results and perform the processing iteratively while varying the filtering and derivative parameters until an optimum result is achieved. It is important to note that at any time in the process, the operator can recover from a processing error, such as distortion as a result of excessive filtering, by simply processing the file again with the proper parameters.

### *Reagents*

Solutions of phenanthrene, anthracene and perylene in cyclohexane were prepared with concentrations in the range of 0·1 to 10 μg/ml by diluting stock 0·01 M solutions with cyclohexane. Aqueous solutions of Pr(III) and Nd(III) were prepared by dissolving the oxides of praseodymium and neodymium in warm hydrochloric acid and diluting with water.

### *Procedure*

The image dissector is adjusted to a 10 kHz bandwidth and scanned between positions corresponding to 181 to 780 nm at a rate of 1000 scans/min. The scans are ensemble averaged to attain an integration time of 16 ms per pixel. Within-run statistics are generated by acquiring three separate spectra with the cuvette and sample being left in the cell holder between spectra. The intensity versus wavelength files are processed for noise reduction using digital filtering algorithms, and first and second derivatives are generated using modified Savitzky-Golay (S–G) convolution functions [7] that generate $(dI/d\lambda)/I$ for first derivatives, or $(d^2I/d\lambda^2)/I$ for second derivatives.

### Results and discussion

All uncertainties are reported as one standard deviation; detection limits are quoted at the 95% confidence level.

### *Spectrophotometer characteristics*

*Wavelength calibration:* The more prominent emission peaks of a mercury penlamp were used for wavelength calibration. For a typical calibration experiment, in which five peaks between 313 and 546 nm were used, a linear least squares fit of wavelength versus pixel number resulted in an intercept of 180·9 ± 0·5 nm, and a slope of 1·160 ± 0·002 nm/pixel, with a standard error of estimate ($S_{yx}$) of 0·26 nm and a correlation coefficient of 0·99999. The statistics indicate excellent linearity near the centre of the scan window. A calibration experiment in which 12 peaks between 254 and 770 nm were used resulted in an intercept of 181·9 ± 0·8 nm and a slope of 1·156 ± 0·004 nm/pixel with a standard error of estimate of 0·90 nm and a correlation coefficient of 0·99997. This is excellent linearity, particularly considering the 600 nm range covered. The specified accuracy of the image dissector is 0·5% of the optical input field, which translates to an absolute error of ±1·48 nm in this configuration. The standard error of estimate indicates that the detector linearity is well within the expected absolute error.

*Dark current:* Dark current was evaluated by averaging 4095 scans with no illumination on the detector. The signal between 180 and 700 nm was always less than the one part/1000 digitization error of the system, demonstrating that the dark current is negligible between 180 and 700 nm. Above 700 nm, the high sensitivity of the image dissector results in the detection of background levels of diffuse radiation resulting in a significant increase of dark current. Subsequent studies were limited to wavelengths shorter than 700 nm.

*Signal-to-noise ratio:* The image dissector is a non-integrating detector and suffers some disadvantages relative to integrating detectors. The signal-to-noise ratio of the image dissector is approximated as:

$$S/N = 1{\cdot}22\, d\, (E\, dt)^{-1/2}$$

where $d$ is the diameter of the aperture (in mils [1·5]), $E$ is the illumination of the face-plate (in foot candles [5–50]), and $dt$ is the dwell time (in μs [4]). A typical value of $S/N$ is about 18 for 25 foot candles of illumination.

A subtle point is that an integrating detector is expected to have an $S/N$ ratio advantage of $N^{1/2}$ with respect to the image dissector, where $N$ is equal to the number of points taken per scan of the device. This poses no serious problem for static (equilibrium) measurements because the fast scanning rate and the negligible dark current permit $S/N$ to be improved by averaging multiple scans. It could present a problem for kinetic studies.

*Linearity:* Solutions of $Pr^{3+}$ and $Nd^{3+}$ were used to evaluate the linearity of response of the spectrophotometer. These ions exhibit sharp absorption bands, resulting in derivative signals that are sensitive indicators of the overall distortion in the system. The results in table 1 indicate that first derivatives computed with a modified Savitzky-Golay smoothing function [7] exhibit a linear dependence on concentration, which is similar to that for absorbance. The small non-zero intercept resulted from contributions to the derivative by the dark current. Results are also given in table 1 for the second derivatives of the $Pr^{3+}$ spectra. The second derivative exhibits a linear dependence on concentration, which is similar to the first derivative. The relative standard deviations for these data are somewhat greater than those for first derivatives, due to the lower $S/N$ ratio of a second-derivative signal.

### *Polynuclear aromatic hydrocarbons*

The quantitation of polynuclear aromatic hydrocarbons (PAH) was used to evaluate the quantitative utility of the image dissector-based spectrophotometer. Intensity versus wavelength data are processed to yield first and second derivatives. Mixtures of PAHs are evaluated using single-wavelength derivative data to achieve quantitative resolution of components.

*Derivative spectra:* Some typical spectra are presented here for illustrative purposes. Figure 1(A) presents the first

*Table 1. Least-squares statistics for first- and second-derivative signals versus concentration of rare-earth ions.*

| Wavelength (nm) | Intercept ± s ($10^{-4}$ ml/mg) | Slope ± s ($10^{-2}$ ml/mg) | Standard error of the estimate ($10^{-3}$ ml/mg) | Correlation coefficient |
|---|---|---|---|---|
| | | First derivatives Pr(III)[a] | | |
| 435 | 2·90 ± 1·3 | 3·22 ± 0·03 | 1·55 | 0·999 |
| 490 | 5·40 ± 1·7 | 2·34 ± 0·07 | 0·35 | 0·999 |
| | | Nd(III)[a] | | |
| 532 | 0·82 ± 2·5 | 6·04 ± 0·07 | 3·31 | 0·999 |
| 575 | 14·9 ± 7·2 | 4·03 ± 0·11 | 9·77 | 0·999 |
| | | Second derivatives Pr(III)[a] | | |
| 435 | 12·7 ± 5·7 | 0·9 ± 0·02 | 0·95 | 0·998 |
| 490 | 18·6 ± 1·3 | 0·8 ± 0·004 | 0·21 | 0·999 |

[a] Five samples, 0·2–5·8 mg/ml.

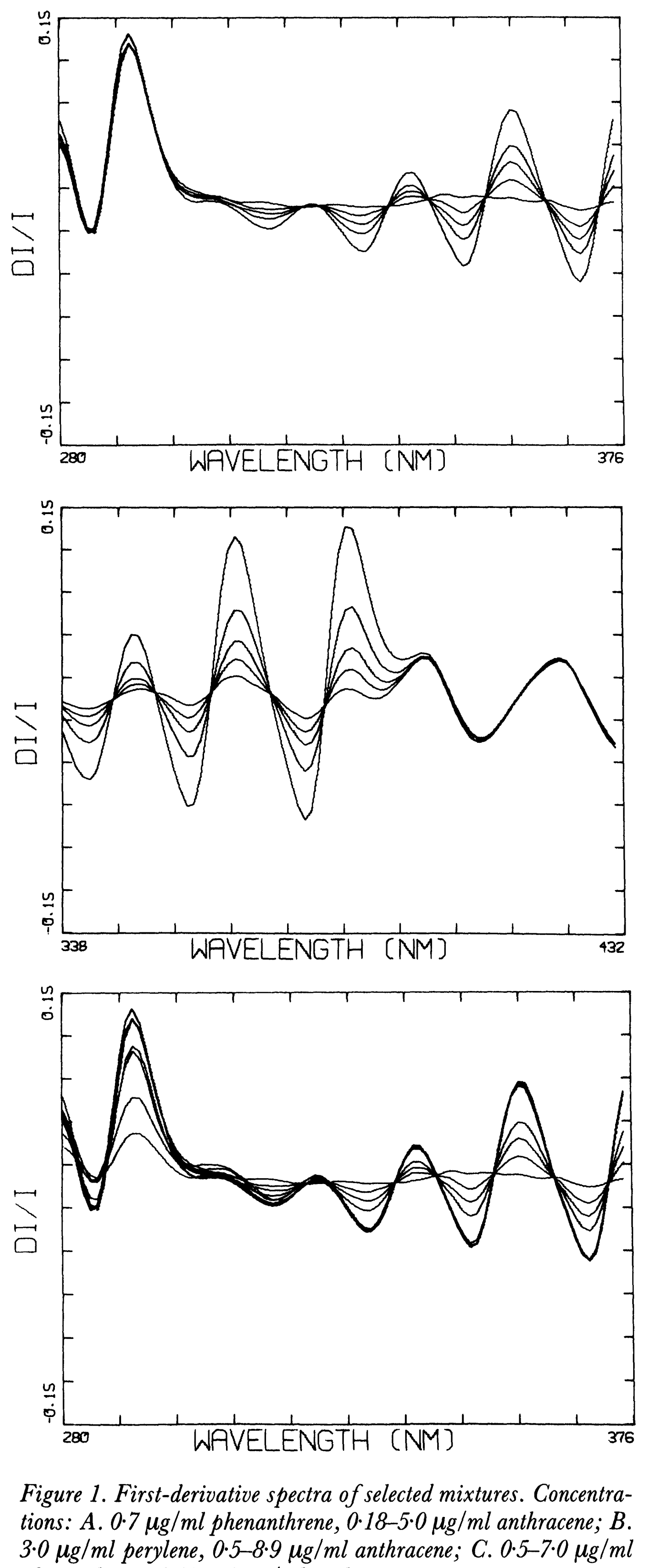

*Figure 1. First-derivative spectra of selected mixtures. Concentrations: A. 0·7 μg/ml phenanthrene, 0·18–5·0 μg/ml anthracene; B. 3·0 μg/ml perylene, 0·5–8·9 μg/ml anthracene; C. 0·5–7·0 μg/ml phenanthrene, 0·18–5·0 μg/ml anthracene.*

derivative spectra of variable concentrations of anthracene with a fixed concentration of phenanthrene, and figure 1(B) presents a similar spectra for anthracene with a fixed concentration of perylene. Other similar spectra with fixed anthracene concentrations and variable amounts of the other species exhibited analogous behavior. These spectra strongly suggest the probability that two- or three-component mixtures of these compounds can be resolved quantitatively. Figure 1(C) shows the spectra of variable concentrations of anthracene and phenanthrene used in quatitative experiments discussed below.

Figures 2(A) and 2(B) show second derivative spectra of solutions with variable concentrations of phenanthrene with a fixed concentration of anthracene, and variable concentrations of anthracene with fixed concentrations of perylene. These and other similar spectra suggest the probability that the second derivative spectra can be used to resolve mixtures quantitatively.

All the spectra presented here have reasonably well resolved isosbestic points. It should be noted that this was not the case with all spectra. In some cases, the instability of the xenon arc lamp caused shifts in spectral positions of a few nanometers. This problem was circumvented in quantitative studies by using a peak-location routine to select derivative peaks.

*Single-component samples:* To establish reference data with which to compare mixture results, different concentra-

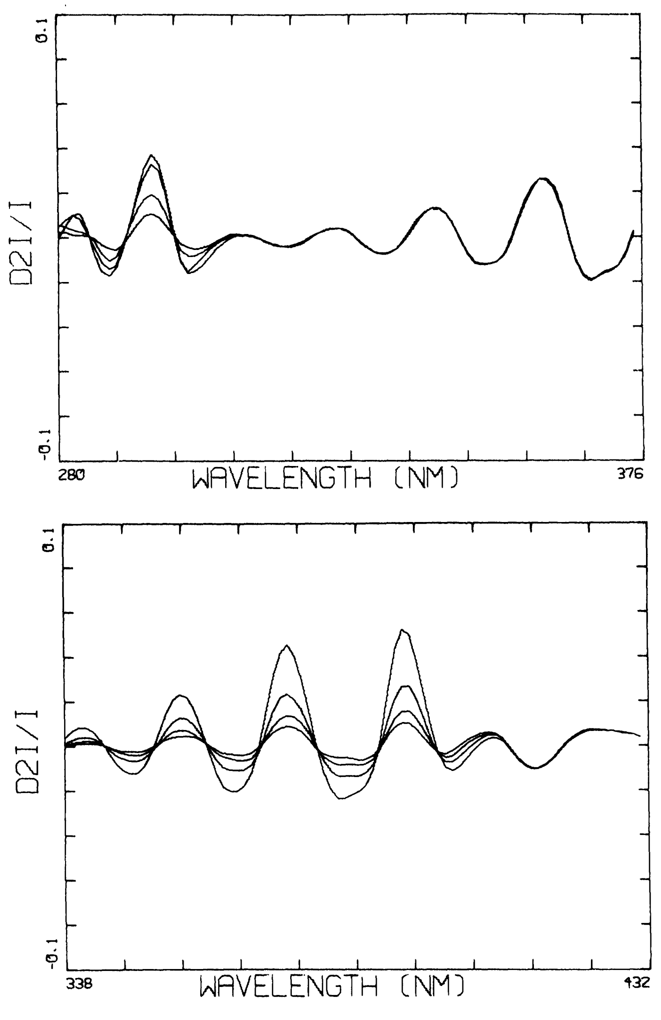

*Figure 2. Second-derivative spectra of selected mixtures. Concentrations: A. 0·4–5·0 μg/ml phenanthrene, 5·0 μg/ml anthracene; B. 3·0 μg/ml perylene, 0·5–7·1 μg/ml anthracene.*

tions of each of the three hydrocarbons were examined; representative results are presented here.

Samples of anthracene with concentrations from 1·0 to 30·0 μg/ml were processed with first derivative data $((dI/d\lambda)/I)$ at 375 nm to determine the linear range of response. The resulting data indicated that the derivative response is linear for concentrations up to 10 μg/ml. Similar linear ranges apply for the other components examined. Least-squares statistics of signal ($y$) versus concentration for anthracene, phenanthrene, and perylene, are given in table 2.

For the first derivative results, the magnitudes of the intercepts are similar to the pooled standard deviations of the results (0·006, 0·19, and 0·04 μg/ml for anthracene, phenanthrene, and perylene, respectively). Using the standard errors and the sensitivities (slopes), the detection limits are estimated to be 0·06, 0·19, and 0·04 μg/ml for anthracene, phenanthrene, and perylene, respectively.

The uncertainties for the phenanthrene standards are about three times greater than the uncertainties for the anthracene and four to five-fold that for perylene; this results because the source intensity and detector response at 290 nm, where the phenanthrene derivatives are determined, are much lower than at 375 at 415nm where anthracene and perylene were quantified.

The spectra were further processed to obtain second derivatives $((d^2I/d\lambda^2)/I))$ and statistical data for the linear least-squares fits of $(d^2I/d\lambda^2)/I$ versus concentration are also given in table 2. The magnitudes of the intercepts are similar to the random uncertainties in the results. The detection limits are estimated to be 0·25, 0·08, and 0·08 μg/ml of phenanthrene, anthracene and perylene respectively. The uncertainties for the second derivatives are 30 to 50% greater than the uncertainties for the first derivatives.

The least-squares statistics indicate excellent linearity for both first and second derivative responses versus concentration.

*Two-component samples:* For the three PAHs discussed above, examination of first and second derivative spectra in the range between 280 and 400 nm (see figures 1 and 2) indicated that there were wavelengths at which individual components could be quantified in the presence of one of the other components. The first three rows of data in table 3 present least-squares statistics for three groups of two-component mixtures quantified with first derivative data. Most of the statistics are similar to those in table 3 for single-component samples, suggesting that the single-wavelength first derivative data can resolve these particular mixtures satisfactorily. The small differences among absolute values of slopes result from the fact that different instrumental parameters were used for different sets of experiments.

For these data, the magnitudes of intercepts are larger than the random uncertainties in results, probably because of interference from the second component. Because of the relative shapes of the spectra, it was expected that second derivative data should reduce this effect. Least-squares statistics for these same samples evaluated with second derivative data are also included in table 3. Detection limits, summarized in the last column of table 3, indicate that the second derivative data are more effective than the first derivative data in reducing the effect of the second component.

*Integration time:* The major source of noise in this experiment is that due to the xenon arc lamp. This was the reason that the uncertainties of measurements for phenanthrene at 290 nm were substantially larger than for the other PAHs. It is expected that the noise from the arc lamp is random and could be reduced by integrating the spectral scans for a longer time. Experiments were performed in which spectra for mixtures of phenanthrene and anthracene were integrated for 16, 64, 256, 512, 1024, and 1536 ms. For these integration times, detection limits for phenanthrene decreased systematically with values of 0·17, 0·083, 0·022, 0·0032, 0·0030, and 0·0029 respectively. These data suggest that integration times up to about 512 ms provide improved performance, beyond that there is little improvement. It is probable that the long-term stability of the lamp limits the effective integration time.

*Table 2. Least-squares statistics for single-component samples of PAHs.*

| Component | Intercept ± s ($10^{-3}$ ml/μg) | Slope ± s ($10^{-2}$ ml/μg) | Standard error of the estimate ($10^{-3}$ ml/μg) | Correlation coefficient |
|---|---|---|---|---|
| | | First derivative | | |
| Anthracene | 6·69 ± 1·05 | 1·50 ± 0·02 | 9·97 | 0·999 |
| Phenanthrene | 5·14 ± 3·18 | 1·55 ± 0·06 | 3·04 | 0·998 |
| Perylene | 8·21 ± 0·73 | 2·24 ± 0·02 | 8·77 | 0·999 |
| | | Second derivative | | |
| Anthracene | 1·82 ± 0·34 | 0·36 ± 0·006 | 3·20 | 0·999 |
| Phenanthrene | −0·72 ± 0·18 | 0·56 ± 0·03 | 1·67 | 0·999 |
| Perylene | 0·22 ± 0·53 | 0·80 ± 0·02 | 6·16 | 0·999 |

Concentration ranges (μg/ml): anthracene, 0·18–5·0; phenanthrene, 0·5–7·0, perylene, 0·4–5·0. Wavelengths for anthracene, phenanthrene, and perylene are bands near 375, 290, and 415 nm, respectively.

*Table 3. Least-squares statistics for two-component mixtures of PAHs. Listed as measured component/background component.*

| Component | Intercept ± s ($10^{-3}$ ml/μg) | Slope ± s ($10^{-2}$ ml/μg) | Standard error of the estimate ($10^{-3}$ ml/μg) | Correlation coefficient | Detection limits (μg/ml) |
|---|---|---|---|---|---|
| | | First derivative | | | |
| Anthracene/ phenanthrene[a] | 3·60 ± 1·85 | 1·43 ± 0·04 | 2·51 | 0·999 | 0·35 |
| Anthracene/ perylene[b] | 5·53 ± 0·56 | 1·34 ± 0·01 | 0·53 | 0·999 | 0·08 |
| Phenanthrene/ anthracene[c] | 2·20 ± 3·47 | 1·59 ± 0·06 | 4·73 | 0·997 | 0·60 |
| | | Second derivative | | | |
| Anthracene/ phenanthrene[a] | 0·772 ± 0·53 | 0·345 ± 0·01 | 0·65 | 0·999 | 0·19 |
| Anthracene/ perylene[b] | −0·255 ± 0·28 | 0·307 ± 0·01 | 0·27 | 0·999 | 0·09 |
| Phenanthrene/ anthracene[c] | 0·063 ± 0·97 | 0·580 ± 0·02 | 1·31 | 0·998 | 0·23 |
| Perylene/ anthracene[d] | 0·704 ± 0·46 | 0·709 ± 0·01 | 0·44 | 0·999 | 0·06 |

[a] 0·18–5·0 μg/ml/0·5–7·0 μg/ml. [b] 0·5–8·9 μ/ml/0·4–5·0 μg/ml. [c] 0·5–7·0 μg/ml/0·18–5·0 μg/ml. [d] 0·4–5·0 μg/0·5–7·0 μg/ml.

Wavelengths used for anthracene, phenanthrene, and perylene are bands near 375, 290, and 415 nm, respectively.

### Discussion

The image dissector has some attractive features for derivative spectroscopy that include positional accuracy of 0·1% or better, very low dark current, and a response that is free of memory and charge migration effects. These characteristics combine to yield fast linear response over three or more orders of magnitude of intensity. On the other hand, it is necessary to use some form of electronic signal averaging to achieve signal-to-noise ratios similar to such integrating detectors as diode arrays.

Although results obtained with this instrumental system are similar to those obtained with diode-array based systems, it is improbable that the image dissector can compete effectively with the diode-array systems for absorbance measurements. However, the high sensitivity of the image dissector makes it particularly attractive for fluorescence measurements. This preliminary study confirms that the image dissector is an effective detector for derivative spectroscopy, and future studies will be focused on luminescence measurements for which the high sensitivity of the image dissector is particularly attractive.

### Acknowledgement

This work was supported by contract DE-ACOZ-79EV10240 from the US Department of Energy.

### References

1. Hager, R. N., *Analytical Chemistry*, **45** (1973), 1131A.
2. O'Haver, T. C., *Analytical Chemistry*, **51** (1979), 91A.
3. Cook, T. E., Santini, R. E. and Pardue, H. L., *Analytical Chemistry*, **49** (1977), 871.
4. Felkel, H. L. and Pardue, H. L., *Analytical Chemistry*, **49** (1977), 1112.
5. Savitzky, A. and Golay, M., *Analytical Chemistry*, **36** (1964), 1628.
6. Bush, I. E., *Analytical Chemistry*, **55** (1983), 2353.
7. Nevius, T. A. and Pardue, H. L., *Analytical Chemistry*, **56** (1984), 2249.

